# Supplementary material for: Depression, anxiety, and stress among frontline health workers during the second wave of COVID-19 in southern Vietnam: A cross-sectional survey
Source: PLOS Glob Public Health. 2022 Sep 8;2(9):e0000823. doi: 10.1371/journal.pgph.0000823 (PMC10022302; doi:10.1371/journal.pgph.0000823)
Supplement: S1 Questionnaire — (DOCX) [file pgph.0000823.s001.docx]

**QUESTIONNAIRE**

**ID: ………………….**

**DEPRESSION, ANXIETY, AND STRESS AMONG FRONTLINE HEALTH WORKERS DURING THE SECOND WAVE OF COVID-19 IN SOUTHERN VIETNAM AND RELATED FACTORS**

*(Please fill out the information and answer (circle) the following questions)*

| **No.** | **Question** | | **Answer** | | | | |
| --- | --- | --- | --- | --- | --- | --- | --- |
| **A** | **GENERAL INFORMATION** | | | | | | |
| **A1** | Abbreviated hospital name: ................................................................................................. | | | | | | |
| **A2** | Study participant initials: …………………………………………………… | | | | | | |
| **A3** | What is your year of birth? | | ……………………….. | | | | |
| **A4** | What is your gender? | | Male | | | | 1 |
|  |  |  | Female | | | | 2 |
| **A5** | What is your ethnicity? | | Kinh | | | | 1 |
|  |  |  | Hoa | | | | 2 |
|  |  |  | Khmer | | | | 3 |
|  |  |  | Other (specify)........................ | | | | 4 |
| **A6** | What is your marital status? | | Single | | | | 1 |
|  |  |  | Marriage | | | | 2 |
|  |  |  | Divorce | | | | 3 |
|  |  |  | Widow | | | | 4 |
|  |  |  | Other (specify)........................ | | | | 5 |
| **A7** | What is your current level of education? | | Intermediate | | | | 3 |
|  |  |  | College | | | | 4 |
|  |  |  | University | | | | 5 |
|  |  |  | Graduate | | | | 6 |
|  |  |  | Other (specify)........................ | | | | 7 |
| **A8** | What is your current working position? | | Doctor | | | | 1 |
|  |  |  | Nursing | | | | 2 |
|  |  |  | Technicians | | | | 3 |
|  |  |  | Cleaners at the hospital | | | | 4 |
|  |  |  | Other (specify)........................ | | | | 5 |
| **A9** | What is your average personal monthly income ?…...……….… million VND | | | | | | |
|  | **Health status** | | | | | | |
| **A10** | How would you assess your current health status?  1. Very good 2. Good 3. Normal 4. Bad 5. Very bad | | | | | | |
| **A11** | How would you assess your own health status at the time of caring for/treating COVID-19 patients in this 2nd wave?  1. Very good 2. Good 3. Normal 4. Bad 5. Very bad | | | | | | |
| **A12** | Your medical history? | | Hypertension | | | 1 | |
|  |  |  | Diabetes | | | 2 | |
|  |  |  | Hyperlipidemia | | | 3 | |
|  |  |  | Cardiovascular disease | | | 4 | |
|  |  |  | Asthma | | | 5 | |
|  |  |  | Mental illness | | | 6 | |
|  |  |  | Other (specify)............... | | | 7 | |
|  |  |  | No disease | | | 8 | |
| **B1** | **Personal factors**  During the hospital receiving care for COVID-19 patients, did you experience any of the following symptoms? | | | | | | |
|  |  | **1. Yes** | | **2. No** | **3. Unclear** | | |
| **B1.1** | Fever ≥38℃ |  | |  |  | | |
| **B1.2** | Sore throat |  | |  |  | | |
| **B1.3** | Runny nose |  | |  |  | | |
| **B1.4** | Cough |  | |  |  | | |
| **B1.5** | Sputum |  | |  |  | | |
| **B1.6** | Trouble breathing |  | |  |  | | |
| **B1.7** | Nausea, vomiting |  | |  |  | | |
| **B1.8** | Diarrhea |  | |  |  | | |
| **B1.9** | Fatigue |  | |  |  | | |
| **B1.10** | Joint pain |  | |  |  | | |
| **B1.11** | Muscle pain |  | |  |  | | |
| **B1.12** | Headache |  | |  |  | | |
| **B1.13** | Insomnia |  | |  |  | | |
| **B1.16** | Loss of appetite |  | |  |  | | |
| **B1.17** | Itching, rash |  | |  |  | | |
| **B1.18** | Other (specify) | ........................................................................ | | | | | |

**SECTION C: Anxiety and Depression Rating Scale (DASS-21)**

| Please read each statement and circle a number 0, 1, 2 or 3 which indicates how much the statement applied to you **over the past week**. There are no right or wrong answers. Do not spend too much time on any statement.  Please read and circle the score in each sentence that is relevant to your situation during this 2nd wave of the COVID-19 pandemic. The rating scale is as follows: 0 Did not apply to me at all 1 Applied to me to some degree, or some of the time 2 Applied to me to a considerable degree or a good part of time 3 Applied to me very much or most of the time |
| --- |

| C1 (s) | I found it hard to wind down | 0 | 1 | 2 | 3 |
| --- | --- | --- | --- | --- | --- |
| C2 (a) | I was aware of dryness of my mouth | 0 | 1 | 2 | 3 |
| C3 (d) | I couldn’t seem to experience any positive feeling at all | 0 | 1 | 2 | 3 |
| C4 (a) | I experienced breathing difficulty (e.g. excessively rapid breathing, breathlessness in the absence of physical exertion) | 0 | 1 | 2 | 3 |
| C5 (d) | I found it difficult to work up the initiative to do things | 0 | 1 | 2 | 3 |
| C6 (s) | I tended to over-react to situations | 0 | 1 | 2 | 3 |
| C7 (a) | I experienced trembling (e.g. in the hands) | 0 | 1 | 2 | 3 |
| C8 (s) | I felt that I was using a lot of nervous energy | 0 | 1 | 2 | 3 |
| C9 (a) | I was worried about situations in which I might panic and make a fool of myself | 0 | 1 | 2 | 3 |
| C10 (d) | I felt that I had nothing to look forward to | 0 | 1 | 2 | 3 |
| C11 (s) | I found myself getting agitated | 0 | 1 | 2 | 3 |
| C12 (s) | I found it difficult to relax | 0 | 1 | 2 | 3 |
| C13 (d) | I felt down-hearted and blue | 0 | 1 | 2 | 3 |
| C14 (s) | I was intolerant of anything that kept me from getting on with what I was doing | 0 | 1 | 2 | 3 |
| C15 (a) | I felt I was close to panic | 0 | 1 | 2 | 3 |
| C16 (d) | I was unable to become enthusiastic about anything | 0 | 1 | 2 | 3 |
| C17 (d) | I felt I wasn’t worth much as a person | 0 | 1 | 2 | 3 |
| C18 (s) | I felt that I was rather touchy | 0 | 1 | 2 | 3 |
| C19 (a) | I was aware of the action of my heart in the absence of physical exertion (e.g. sense of heart rate increase, heart missing a beat) | 0 | 1 | 2 | 3 |
| C20 (a) | I felt scared without any good reason | 0 | 1 | 2 | 3 |
| C21 (d) | I felt that life was meaningless | 0 | 1 | 2 | 3 |

**SECTION E. RELATED FACTORS**

| **No.** | **Question** | **Answer** | | | |
| --- | --- | --- | --- | --- | --- |
| **E2** | **Personal knowledge about COVID-19** |  | | | |
| E2.1 | Do you believe that protective gear can prevent COVID-19 infection?  1. Very trusting 2. Trusting 3. Normal 4. Not trusting 5. Very distrustful | | | | |
| E2.2 | Are you fear of transmitting COVID-19 to family and friends?  1. Very worried 2. Worried 3. Normal 4. Not worried 5. Very not worried | | | | |
| **E3** | **Family and social factors** | | | | |
| E3.1 | Do you have relatives or friends infected with COVID-19? | | 1. Yes           2. No | | |
| E3.2 | Have you been shunned or discriminated against by your friends, family, or community because of being a health worker? | | 1. Yes           2. No | | |
| E3.3 | Is your family shunned or discriminated against by the community because you are a health worker? | | 1. Yes           2. No | | |
| E3.4 | Do you receive spiritual and material support from friends, family, and the community? | | 1. Yes           2. No | | |
| E3.5 | Do you feel anxious every time you see information in the media about COVID-19? | | 1. Yes           2. No | | |
| **E4** | **Occupational factors and working environment** | | | | |
| E4.1 | In which department do you work? | ..……………………… | | | |
| E4.2 | How many years of work do you currently have in the hospital? | …...... month or.........year | | | |
| E4.3 | On average, how many hours did you work a day before COVID-19? | …..... hour/day | | | |
| E4.4 | How many hours per day on average do you work during this 2nd wave of COVID-19? | …..... hour/day | | | |
| E4.5 | How would you assess the amount of work to be done during the COVID-19 pandemic?  1. Very much 2. Much 3. Normal 4. Little 5. Very little | | | | |
| E4.6 | Have you been tested for COVID-19 while on the job? | 1. Yes           2. No | | | |
| E4.7 | Have you ever had to quit your job to go to concentrated isolation? | 1. Yes           2. No  **(Do not 🡪 move to E4.9)** | | | |
| E4.8 | If quarantined, what worries you during quarantine? (multiple options) | orried about being infected with COVID-19 | | | 1 |
|  |  | Worried about infecting relatives and friends | | | 2 |
|  |  | Worried that no one will take care of the children/relatives | | | 3 |
|  |  | Worried that the family economy will be affected | | | 4 |
|  |  | Fear of being shunned and stigmatized | | | 5 |
|  |  | Other (specify) :...............  ................................................. | | | 6 |
| E4.9 | Have you received psychological counseling and support during the care and treatment of COVID-19 patients? | 1. Yes           2. No | | | |
| E4.10 | Do you have a shortage of protective gear while caring for a COVID-19 patient? | 1. Yes           2. No | | | |
| E4.11 | Does the hospital where you work have adequate equipment to treat COVID-19 patients? | 1. Yes           2. No | | | |
| E4.12 | Did you receive infection control training before caring for and treating COVID-19 patients? | 1. Yes           2. No | | | |
| E4.13 | How would you assess your own compliance practices for infection prevention measures?1. 1. Very good 2. Good 3. Normal 4. Not good 5. Very not good | | | | |
| E4.14 | How often do you wash your hands while caring for and treating COVID-19 patients?  1. Always 2. Often 3. Sometimes 4. Rarely 5. Never | | | | |
| E4.15 | Have you been in direct contact with a patient with COVID-19? | | 1. Yes           2. No  **(Do not 🡪 move to E4.7)** | | |
| E4.16 | How many hours in a day do you come into direct contact with a patient with COVID-19? | | ………………..hour | | |
| E4.17 | How would you assess yourself wearing protective gear while caring for a COVID-19 patient??  1. Always 2. Often 3. Sometimes 4. Rarely 5. Never | | | | |
| E4.18 | How often have you been in contact with secretions of COVID-19 patients? | | 1. Yes           2. No  **(Do not 🡪 move to F)** | | |
| E4.19 | What kind of secretions of COVID-19 patients are you usually exposed to? | | Blood | 1 | |
|  |  |  | Sputum | 2 | |
|  |  |  | Urine | 3 | |
|  |  |  | Other (specify):.............. | 4 | |

**F**. Finally, in addition to the questions asked above, is there anything that makes you worry (stress) during the time of caring for and treating patients with COVID-19?

……………………………………………………………………………………………………………………………………………………………………………………………………………………………..

……………………………………………………………………………………………………………..

……………………………………………………………………………………………………………..

***Thank you for participating in the study!***
