# Supplementary material for: Depression, anxiety, and stress among frontline health workers during the second wave of COVID-19 in southern Vietnam: A cross-sectional survey
Source: PLOS Glob Public Health. 2022 Sep 8;2(9):e0000823. doi: 10.1371/journal.pgph.0000823 (PMC10022302; doi:10.1371/journal.pgph.0000823)
Supplement: S2 Questionnaire — (DOCX) [file pgph.0000823.s002.docx]

**BỘ CÂU HỎI PHỎNG VẤN**

**ID: ………………….**

**STRESS, LO ÂU VÀ TRẦM CẢM CỦA NHÂN VIÊN Y TẾ TUYẾN ĐẦU TRONG**

**ĐẠI DỊCH COVID-19 THỨ 2 TẠI CÁC BỆNH VIỆN KHU VỰC PHÍA NAM, VIỆT NAM VÀ**

**CÁC YẾU TỐ LIÊN QUAN**

Anh/chị vui lòng điền thông tin và trả lời (khoanh tròn) các câu hỏi dưới đây:

| **STT** | **Câu hỏi** | | **Trả lời** | | | | |
| --- | --- | --- | --- | --- | --- | --- | --- |
| **A** | **THÔNG TIN CHUNG** | | | | | | |
| **A1** | Tên bệnh viện:................................................................................................. | | | | | | |
| **A2** | Họ tên người được phỏng vấn:…………………………………………………… | | | | | | |
| **A3** | Năm sinh của anh/chị? | | ……………………….. | | | | |
| **A4** | Giới tính của anh/chị? | | Nam | | | | 1 |
|  |  |  | Nữ | | | | 2 |
| **A5** | Dân tộc của anh/chị? | | Kinh | | | | 1 |
|  |  |  | Hoa | | | | 2 |
|  |  |  | Khơ Me | | | | 3 |
|  |  |  | Khác (ghi rõ).......................... | | | | 4 |
| **A6** | Tình trạng hôn nhân của anh/chị là gì? | | Độc thân | | | | 1 |
|  |  |  | Kết hôn | | | | 2 |
|  |  |  | Li dị | | | | 3 |
|  |  |  | Góa | | | | 4 |
|  |  |  | Khác (ghi rõ).......................... | | | | 5 |
| **A7** | Trình độ học vấn của anh/chị hiện tại? | | Trung học | | | | 3 |
|  |  |  | Cao đẳng | | | | 4 |
|  |  |  | Đại học | | | | 5 |
|  |  |  | Sau đại học | | | | 6 |
|  |  |  | Khác (ghi rõ).......................... | | | | 7 |
| **A8** | Vị trí làm việc của anh/chị hiện tại? | | Bác sĩ | | | | 1 |
|  |  |  | Điều dưỡng | | | | 2 |
|  |  |  | Kỹ thuật viên | | | | 3 |
|  |  |  | Hộ lý | | | | 4 |
|  |  |  | Khác (ghi rõ).......................... | | | | 5 |
| **A9** | Thu nhập bình quân cá nhân anh/chị hàng tháng là bao nhiêu? …...……….… triệu đồng | | | | | | |
|  | **Tình trạng sức khỏe** | | | | | | |
| **A10** | Anh/chị tự đánh giá tình trạng sức khỏe hiện tại của mình ở mức nào?  1. Rất tốt 2. Tốt 3. Bình thường 4. Kém 5. Rất kém | | | | | | |
| **A11** | Anh/chị tự đánh giá tình trạng sức khỏe của mình ở thời điểm chăm sóc/điều trị bệnh nhân COVID-19 mức nào?  1. Rất tốt 2. Tốt 3. Bình thường 4. Kém 5. Rất kém | | | | | | |
| **A12** | Tiền sử bệnh lý của anh/chị? | | Tăng huyết áp | | | 1 | |
|  |  |  | Đái tháo đường | | | 2 | |
|  |  |  | Tăng lipid máu | | | 3 | |
|  |  |  | Bệnh tim mạch | | | 4 | |
|  |  |  | Hen suyễn | | | 5 | |
|  |  |  | Bệnh lý tâm thần | | | 6 | |
|  |  |  | Bệnh khác (ghi rõ:..........  ………………………...) | | | 7 | |
|  |  |  | Không có bệnh lý | | | 8 | |
| **B1** | **Yếu tố cá nhân**  Trong thời gian bệnh viện tiếp nhận chăm sóc điều trị bệnh nhân COVID-19 anh/chị có bị các triệu chứng nào sau đây không: | | | | | | |
|  |  | **1. Có** | | **2. Không** | **3. Không rõ** | | |
| **B1.1** | Sốt ≥38℃ |  | |  |  | | |
| **B1.2** | Đau, rát họng |  | |  |  | | |
| **B1.3** | Chảy nước mũi |  | |  |  | | |
| **B1.4** | Ho |  | |  |  | | |
| **B1.5** | Đờm |  | |  |  | | |
| **B1.6** | Khó thở |  | |  |  | | |
| **B1.7** | Buồn nôn, nôn |  | |  |  | | |
| **B1.8** | Tiêu  chảy |  | |  |  | | |
| **B1.9** | Mệt mỏi |  | |  |  | | |
| **B1.10** | Đau khớp |  | |  |  | | |
| **B1.11** | Đau cơ |  | |  |  | | |
| **B1.12** | Đau đầu |  | |  |  | | |
| **B1.13** | Mất ngủ |  | |  |  | | |
| **B1.16** | Ăn mất ngon |  | |  |  | | |
| **B1.17** | Ngứa, phát ban |  | |  |  | | |
| **B1.18** | Khác (ghi rõ) | ........................................................................ | | | | | |

**PHẦN C: THANG ĐÁNH GIÁ LO ÂU TRẦM CẢM (DASS-21)**

| Dưới đây là những biểu hiện tâm lý thường thấy. Hãy đọc kỹ từng câu và khoanh tròn **MỘT** số thích hợp biểu thị đúng nhất trạng thái tâm lý của bạn trong **thời gian đại dịch COVID- 19 đang xảy ra**. | | | | | |
| --- | --- | --- | --- | --- | --- |
| Xin anh/chị vui lòng đọc và khoanh tròn số điểm số ở từng câu phù hợp với tình trạng gặp phải trong thời gian đại dịch COVID-19.  **0 Điều này hoàn toàn không xảy ra cho Tôi**  **1 Xảy ra cho Tôi một phần nào, hay thỉnh thoảng**  **2 Thường xảy ra cho Tôi, hay nhiều lần**  **3 Rất thường xảy ra, hay hầu hết lúc nào cũng có** | | | | | |
| **TT** | **Câu hỏi** | **Cho điểm** | | | |
| **C1** | Anh/chị nhận thấy khó mà nghỉ ngơi | 0 | 1 | 2 | 3 |
| **C2** | Anh/chị thấy mình bị khô miệng | 0 | 1 | 2 | 3 |
| **C3** | Anh/chị không thấy có một cảm giác lạc quan nào  cả | 0 | 1 | 2 | 3 |
| **C4** | Anh/chị bị khó thở (thở nhanh, khó thở mà không do làm việc mệt) | 0 | 1 | 2 | 3 |
| **C5** | Anh/chị thấy khó mà bắt tay vào làm công việc | 0 | 1 | 2 | 3 |
| **C6** | Anh/chị đã phản ứng thái quá khi có những sự việc xảy ra | 0 | 1 | 2 | 3 |
| **C7** | Tay anh/chị bị run | 0 | 1 | 2 | 3 |
| **C8** | Anh/chị thấy mình đã tốn quá nhiều năng lượng cho việc lo lắng | 0 | 1 | 2 | 3 |
| **C9** | Anh/chị lo mình đến những nơi mà bạn có thể bị hốt hoảng và tự làm mất mặt | 0 | 1 | 2 | 3 |
| **C10** | Anh/chị thấy tương lai mình chả có gì để mong chờ | 0 | 1 | 2 | 3 |
| **C11** | Anh/chị thấy bồn chồn | 0 | 1 | 2 | 3 |
| **C12** | Anh/chị thấy khó mà thư giãn | 0 | 1 | 2 | 3 |
| **C13** | Anh/chị thấy mình xuống tinh thần và buồn rầu | 0 | 1 | 2 | 3 |
| **C14** | Anh/chị thấy thiếu kiên nhẫn với những điều cản trở việc bạn đang làm | 0 | 1 | 2 | 3 |
| **C15** | Anh/chị thấy mình gần như bị hốt hoảng | 0 | 1 | 2 | 3 |
| **C16** | Anh/chị không thấy hăng hái để làm bất cứ chuyện gì | 0 | 1 | 2 | 3 |
| **C17** | Anh/chị thấy mình là người kém giá trị | 0 | 1 | 2 | 3 |
| **C18** | Anh/chị thấy mình rất dễ nhạy cảm | 0 | 1 | 2 | 3 |
| **C19** | Anh/chị thấy tim mình đập nhanh, đập hụt nhịp mà không do làm việc mệt | 0 | 1 | 2 | 3 |
| **C20** | Anh/chị cảm thấy sợ vô cớ | 0 | 1 | 2 | 3 |
| **C21** | Anh/chị cảm thấy cuộc sống mình không có ý nghĩa | 0 | 1 | 2 | 3 |

**PHẦN E. KIẾN THỨC VỀ COVID-19 VÀ CÁC YẾU TỐ LIÊN QUAN ĐẾN LO ÂU TRẦM CẢM**

| **TT** | **Câu hỏi** | **Trả lời** | | | |
| --- | --- | --- | --- | --- | --- |
| **E2** | **Kiến thức cá nhân về COVID-19** |  | | | |
| E2.1 | Anh/chị có tin tưởng đồ bảo hộ có thể phòng ngừa lây nhiễm COVID-19 không?  1. Rất tin tưởng 2. Tin tưởng 3. Bình thường 4. Không tin tưởng 5. Rất không tin tưởng | | | | |
| E2.2 | Anh/chị có lo lắng bản thân lây nhiễm COVID-19 cho gia đình và bạn bè không?  1. Rất lo lắng 2. Lo lắng 3. Bình thường 4. Không lo lắng 5. Rất không lo lắng | | | | |
| **E3** | **Yếu tố gia đình - xã hội** | | | | |
| E3.1 | Anh/chị có người thân, bạn bè mắc COVID-19 không? | | 1. Có            2. Không | | |
| E3.2 | Anh/chị có bị bạn bè, gia đình, cộng đồng xa lánh, kì thị vì là nhân viên y tế không? | | 1. Có            2. Không | | |
| E3.3 | **Gia đình** anh/chị có bị cộng đồng xa lánh, kỳ thị vì anh/chị là nhân viên y tế không? | | 1. Có            2. Không | | |
| E3.4 | Anh/chị có nhận được sự hỗ trợ tinh thần, vật chất từ bạn bè, gia đình, cộng đồng không? | | 1. Có            2. Không | | |
| E3.5 | Anh/chị có cảm thấy lo lắng mỗi khi xem các thông tin trên truyền thông về COVID-19 không? | | 1. Có            2. Không | | |
| **E4** | **Yếu tố nghề nghiệp và môi trường làm việc** | | | | |
| E4.1 | Anh/chị làm việc ở khoa phòng nào? | ..……………………… | | | |
| E4.2 | Số năm công tác của anh/chị trong bệnh viện hiện tại? | …...... tháng hoặc.........năm | | | |
| E4.3 | Anh/chị làm việc trung bình bao nhiêu giờ 1 ngày  **trước** dịch COVID-19? | …..... giờ/ngày | | | |
| E4.4 | Anh/chị làm việc trung bình bao nhiêu giờ 1 ngày **trong** dịch COVID-19? | ….....giờ/ngày | | | |
| E4.5 | Anh/chị đánh giá như thế nào về khối lượng công việc phải làm trong thời gian đại dịch COVID-19?  1. Rất nhiều 2. Nhiều 3. Bình thường 4. Ít 5. Rất ít | | | | |
| E4.6 | Anh/chị có được xét nghiệm COVID-19 trong quá trình làm việc không? | 1. Có            2. Không | | | |
| E4.7 | Anh/chị có bao giờ phải nghỉ việc để đi cách ly tập trung không? | 1. Có            2. Không  **(Không chuyển E4.9)** | | | |
| E4.8 | Nếu có bị cách ly, điều gì khiến anh/chị lo lắng trong thời gian bị cách ly (nhiều lựa chọn) | Lo bị nhiễm bệnh covid-19 | | | 1 |
|  |  | Lo lây nhiễm cho người thân, bạn bè | | | 2 |
|  |  | Lo không ai chăm sóc con cái/người thân | | | 3 |
|  |  | Lo kinh tế gia đình bị ảnh hưởng | | | 4 |
|  |  | Lo bị xa lánh, kỳ thị | | | 5 |
|  |  | Khác (ghi rõ):.....................  ................................................. | | | 6 |
| E4.9 | Anh/chị có được tư vấn và hỗ trợ về tâm lý trong quá trình chăm sóc và điều trị bệnh nhân COVID-19 không? | 1. Có            2. Không | | | |
| E4.10 | Anh/chị có bị thiếu đồ bảo hộ trong thời gian chăm sóc bệnh nhân COVID-19? Bộ bảo hộ gồm áo, quần, giày, bao tay, khẩu trang, kính, mặt nạ. | 1. Có            2. Không | | | |
| E4.11 | Cơ sở y tế của anh/chị có đầy đủ trang thiết bị điều trị cho bệnh nhân COVID-19 không? | 1. Có            2. Không | | | |
| E4.12 | Anh/chị có được đào tạo phòng ngừa kiểm soát nhiễm khuẩn trước khi chăm sóc và điều trị bệnh nhân COVID-19 không? | 1. Có            2. Không | | | |
| E4.13 | Anh/chị tự đánh giá về thực hành tuân thủ của bản thân về các biện pháp phòng ngừa lây nhiễm như thế nào?  1. Rất tốt 2. Tốt 3. Bình thường 4. Không tốt 5. Rất không tốt | | | | |
| E4.14 | Anh/chị đánh giá mức độ thường xuyên rửa tay của bản thân trong thời gian chăm sóc, điều trị bệnh nhân COVID-19?  1. Luôn luôn 2. Thường xuyên 3. Đôi khi 4. Hiếm khi 5. Không bao giờ | | | | |
| E4.15 | Anh/chị có tiếp xúc trực tiếp với bệnh nhân nhiễm COVID-19 không? | | 1. Có            2. Không  **(Không🡪 chuyển E4.17)** | | |
| E4.16 | Anh/chị tiếp xúc trực tiếp với bệnh nhân nhiễm COVID-19 bao nhiêu giờ trong 1 ngày | | ……………….. giờ | | |
| E4.17 | Anh/chị đánh giá mức độ mặc đồ bảo hộ của bản thân trong thời gian chăm sóc bệnh nhân COVID-19?  1. Luôn luôn 2. Thường xuyên 3. Đôi khi 4. Hiếm khi 5. Không bao giờ | | | | |
| E4.18 | Anh/chị thường xuyên tiếp xúc với dịch tiết nào của bệnh nhân COVID-19 không? | | 1. Có             2. Không **(Không🡪 chuyển F)** | | |
| E4.19 | Anh/chị thường tiếp xúc với loại dịch tiết nào của bệnh nhân COVID-19? | | Máu | 1 | |
|  |  |  | Đờm | 2 | |
|  |  |  | Nước tiểu | 3 | |
|  |  |  | Khác (ghi rõ): .......... | 4 | |

**F**. **Cuối cùng,** ngoài những vấn đề đã được hỏi trên, còn điều gì khiến anh/chị phải lo lắng (stress) trong thời gian chăm sóc và điều trị bệnh nhân mắc COVID-19?................................................................

……………………………………………………………………………………………………………………………………………………………………………………………………………………………..

……………………………………………………………………………………………………………..

……………………………………………………………………………………………………………..

***Cảm ơn anh/chị đã tham gia phỏng vấn!***
