# Supplementary material for: Depression, anxiety, and stress among frontline health workers during the second wave of COVID-19 in southern Vietnam: A cross-sectional survey
Source: PLOS Glob Public Health. 2022 Sep 8;2(9):e0000823. doi: 10.1371/journal.pgph.0000823 (PMC10022302; doi:10.1371/journal.pgph.0000823)
Supplement: Attachment — Submitted filename: PLOS GH DASS VN revisions 26 Apr 2021.docx [file pgph.0000823.s004.docx]

06 MAY 2022

Dear Editors,

On behalf of Ms Anh Le Thi Ngoc and other co-authors, attached find the revised article entitled “Depression, anxiety, and stress among frontline health workers during the second wave of COVID-19 in southern Vietnam: a cross-sectional survey” for re-review in *PLOS Global Public Health*.

Editor comments:

1. Participant consent: added that consent was “informed” on page 6
2. Survey added as supplemental information in both English and Vietnamese
3. Added new figure files in the appropriate format - .tiff
4. We will add the dataset to the University of Oxford repository
5. Financial disclosure statement edited accordingly

Reviewer #1:

Thank you for the many relevant references. We enriched the introduction and added some of these key references to our paper. See attached.

Reviewer #2:

Thank you for the suggestion to revise the introduction to include more literature. This was also suggested by Reviewer #1 so we enriched the introduction and added some of these key references to our paper.

Reviewer #3: Thank you for the interesting points and useful references. We added these and other literature to the introduction (see also reviewer 1 and 2 responses).

Reviewer #4:

1. Revised the paragraph as suggested, thank you.
2. The ethics clearance is listed in lines 149-150.
3. Thank you for mentioning this point. Before conducting the research, we sent dispatches to 14 hospitals treating COVID-19 patients in the southern region of Vietnam. We clearly stated the purpose of the study. Our Institute of Public Health has the function of managing 20 provinces in the Southern Region of Vietnam in disease prevention, so the hospital directors and department managers where we had planned to collect the data agreed and coordinated very well.

About preventive measures: When our team went to the hospital to collect data, we all made a medical declaration, disinfected, kept distance when contacting staff, and wore masks. For health workers at the time we came to collect the data, they did not have to be isolated in the COVID-19 patient treatment area because they had just completed their tasks in this area. We gathered with them in a hospital office (with disinfection, physical distancing when contacting and wearing masks) to collect data. For the health workers who were already in the isolation area treating COVID-19 patients, we were supported by hospital staff to send research information, consent forms and questionnaires for data collection and they sent it back to us.

We added a bit of this detailed information to lines 120-122 for clarification.

4. Added that survey was in Vietnamese language to line 102
5. Lines 113 – Added references
6. Edited to Stata 13.0
7. Added more specifics to line 90.
8. Thank you.
9. Added information about the study contribution at lines 331-333.

Reviewer #5: Thank you for your comments.

Please let us know if you need further information.

With best wishes,

**Jennifer Ilo Van Nuil, PhD**

**Research Fellow**

Centre for Tropical Medicine

Oxford University Clinical Research Unit

764 Vo Van Kiet, W.1, Dist.5, Ho Chi Minh City

Vietnam

Wellcome Trust Asia Programme

In partnership with

Hospital for Tropical Diseases

Mob: 84 (0) 36262 0124

E-mail: [jvannuil@oucru.org](mailto:jvannuil@oucru.org)<<mailto:jvannuil@oucru.org>>

Website: [http://oucru.org](http://oucru.org/)
